# Exploring Behaviors of Caterpillar-Like Soft Robots with a Central Pattern Generator-Based Controller and Reinforcement Learning

**DOI:** 10.1089/soro.2018.0126

**Published:** 2019-10-04

**Authors:** Matthew Ishige, Takuya Umedachi, Tadahiro Taniguchi, Yoshihiro Kawahara

**Affiliations:** ^1^ Graduate School of Information Science and Technology, The University of Tokyo, Bunkyo-ku, Tokyo, Japan.; ^2^ Department of Information Science and Engineering, Ritsumeikan University, Kusatsu, Shiga, Japan.

**Keywords:** caterpillar-like soft-bodied robots, gait controller, mechanosensory feedback, central pattern generator, policy gradients with parameter-based exploration

## Abstract

Due to their flexibility, soft-bodied robots can potentially achieve rich and various behaviors within a single body. However, to date, no methodology has effectively harnessed these robots to achieve such diverse desired functionalities. Controllers that accomplish only a limited range of behaviors in such robots have been handcrafted. Moreover, the behaviors of these robots should be determined through body–environment interactions because an appropriate behavior may not always be manifested even if the body dynamics are given. Therefore, we have proposed SenseCPG-PGPE, a method for automatically designing behaviors for caterpillar-like soft-bodied robots. This method optimizes mechanosensory feedback to a central pattern generator (CPG)-based controller, which controls actuators in a robot, using policy gradients with parameter-based exploration (PGPE). In this article, we deeply investigated this method. We found that PGPE can optimize a CPG-based controller for soft-bodied robots that exhibit viscoelasticity and large deformation, whereas other popular policy gradient methods, such as trust region policy optimization and proximal policy optimization, cannot. Scalability of the method was confirmed using simulation as well. Although SenseCPG-PGPE uses a CPG-based controller, it can achieve nonsteady motion such as climbing a step in a simulated robot. The approach also resulted in distinctive behaviors depending on different body–environment conditions. These results demonstrate that the proposed method enables soft robots to explore a variety of behaviors automatically.

## Introduction

Bioinspired soft-bodied robots^1^ should be controlled in a bioinspired manner. Conventional control schemes are not applicable to soft-bodied robots because these robots have considerably more degrees of freedom due to their significant flexibility. Moreover, designing behaviors themselves is challenging because body–environment interactions involve complicated dynamics (e.g., surface friction switching). As Corucci *et al.*^2^ demonstrate, both body morphology and environmental factor affect the optimal behavior of soft-bodied robots. Instead, a central pattern generator (CPG)-based controller, which is a bioinspired control method based on parts of the neural system in animals,^3^ is a promising candidate to harness and direct the complexity of these robots. This control method does not face the above-mentioned issues because it does not demand the precise design of a movement trajectory.^4–8^ It also invokes automatic behavior switching according to the body and environment dynamics.^9,10^ Owaki and Ishiguro^10^ realized automatic switching of gait patterns among walking, trotting, and galloping in a quadruped robot with a sensor feedback integrated CPG-based controller. A CPG-based controller with sensor feedback was applied to a caterpillar-like soft-bodied robot as well.^11^

However, how to integrate and tune sensor feedback in a CPG-based controller, especially for soft-bodied robots, remains an open issue.^10–13^ Because soft-bodied robots have large degrees of freedom, each actuator should be controlled using data from many sensors throughout the body, so that they coordinate to achieve the desired behavior of the robot as a whole. This requirement leads to parameter tuning among an enormous number of sensor–actuator combinations. Furthermore, the number of sensors and actuators available will increase as the implementation of more sensors and actuators becomes possible with the advancement of digital fabrication and automation technologies. Tuning parameters in such a situation is a difficult task for robot designers. Even if a controller could be handcrafted, it would only be able to explore a limited space of behaviors and is not scalable across various robots and environments.

To tune a large number of sensor feedback parameters for a soft-bodied robot systematically and efficiently, we^14^ proposed the utilization of policy gradients with parameter-based exploration (PGPE),^15^ which is an episode-based reinforcement learning method. Previous work^16–18^ has specifically applied reinforcement learning to tune sensor feedback for CPG-based controllers. Matsubara *et al.*^17^ tuned sensor feedback for a CPG-based controller of a rigid-bodied biped robot using an actor–critic algorithm. They managed to converge training within relatively fewer iterations by resourcefully reducing policy exploration space with auxiliary heuristics on walking behavior. However, as this study aimed to explore behavior automatically, we excluded such heuristics on behaviors. Furthermore, we previously found that an actor–critic algorithm does not function in a system where uncontrollable and unobservable degrees of freedom exist, as in soft-bodied robots.^14^ Therefore, PGPE is considered a suitable candidate for optimizing a CPG-based controller in a soft-bodied robot. By automatically tuning feedback to a CPG-based controller, distinctive behaviors are acquired under different body and environment conditions. Even parameters for nonsteady motions, such as climbing a step, can be tuned effortlessly.

In this study we investigate the effectiveness of SenseCPG-PGPE, a method we previously proposed^14^ to design behaviors of caterpillar-like soft-bodied robots. We show several important properties of this method. First, it can scale to design controllers for robots with high numbers of sensors and actuators. This is necessary for manipulating increasingly complex soft-bodied robots which will become available as technologies advance. Second, it has the ability to achieve nonsteady motion even though it uses a CPG-based controller. Hence, we can use the method to accomplish not only periodical steady motion but also more complex behaviors in soft-bodied robots. Finally, we show that the method creates different controllers that generate distinctive behaviors, such as crawling and inching in a caterpillar-like robot, under different body and environmental dynamics. This result implies that we can fully exploit the flexibility of soft-bodied robots to achieve diverse behaviors using the proposed method. Although we have focused on caterpillar-like morphology, the ultimate goal of this research is to construct a general framework for manipulating soft-bodied robots. The introduction of PGPE enables controllers to acquire knowledge about morphology and environment a posteriori, creating adaptive behaviors in a complex real world. We believe that this advances soft robotics by allowing flexible soft-bodied robots to operate by skillfully navigating their environment. We consider caterpillars to be a good starting point for this goal because they can exhibit a wide variety of behaviors despite their morphological simplicity. We reproduced behaviors of such caterpillars in a computer by likewise applying a simple method, that is, optimizing sensor feedback to the CPG-based controller using PGPE.

The remainder of this article is organized as follows. Controller Building Framework section discloses details of SenseCPG-PGPE. Mechanical System section describes the simulation model used to validate the ability of the framework. Experiments section presents numerical simulations and their results. Finally, Discussion section presents analysis and discussion on the method.

## Controller Building Framework

This section shares the structure of SenseCPG-PGPE. First, we introduce the CPG-based controller and explain how it is used to control a soft-bodied robot. Then we describe in detail the oscillators used in the CPG-based controller and the optimization method. Finally, we describe the overall mechanism.

### CPG-based controller for caterpillar-like soft robots

**FIG. 1.**
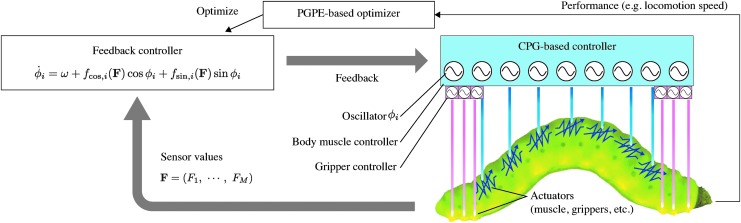
Overview of SenseCPG-PGPE. Displacement distance or angle of each actuator in a control objective (depicted here as a caterpillar-like robot) obeys the phase of an active rotator in the CPG-based controller. The feedback controller receives the mechanosensor values obtained across the body and calculates feedback to the CPG-based controller according to the equation. After running this cycle for several steps, the PGPE algorithm updates the parameters in the feedback controller based on performance. CPG, central pattern generator; PGPE, policy gradients with parameter-based exploration.

As shown in Figure 1, a CPG-based controller controls a caterpillar-like soft-bodied robot in the proposed method. The CPG-based controller is modeled by means of phase oscillators, where each oscillator drives an associated actuator. The displacement of each actuator is directly related to the corresponding oscillator phase (see Mechanical System section). In many CPG-based controllers, oscillators are directly coupled, and the coupling coefficients are tuned manually. Instead, we do not explicitly build these connections, and let the oscillators communicate only through mechanosensory feedback.

### Active rotator for mechanosensor integration

A CPG-based controller receives feedback from mechanosensors to interact with the environment. To feed mechanosensor values to a phase oscillator, we adopted an active rotator^19^ instead of the usual phase oscillator. An active rotator is expressed as

\begin{align*}
\dot \phi = \omega - X \sin ( \phi - \alpha ) ,
\end{align*}

where $\dot \phi$ is the phase of the oscillator, *ω* is a nominal angular velocity, and *X* is a non-negative value. This is a normal phase oscillator when *X* = 0, whereas $\dot \phi$ is attracted to a constant phase $\alpha + \arcsin \frac { w } { X }$ when *X* is positive. Reflex can be embedded by replacing *X* with a sensor value. Owaki and Ishiguro^10^ succeeded in generating gait pattern transition by replacing *X* with the force on the soles. In this study, the rotator is extended to utilize all mechanosensors over the body. The rule for the *i*-th oscillator is

\begin{align*}
{ \dot \phi _i} = \omega + {f_{ \cos , i}} ( {\bf F} ) \cos { \phi _i} + {f_{ \sin , i}} ( {\bf F} ) \sin { \phi _i} , \tag{1}
\end{align*}

where **F** = (*F*_1_, …, *F_M_*) is a vector of mechanosensor values, and *f*_cos,*i*_(**F**) and *f*_sin,*i*_(**F**) are arbitrary linear functions that receive the mechanosensor values **F** to calculate the scalar values.

\begin{align*}
{f_{ \cos , i}} ( {\bf F} ) = \max \{ \min \{ {f_{ \max }} , { ( {W_{ \cos }}{ \bf F} + {{ \bf b}_{ \cos }} ) _i} \} , - {f_{ \max }} \} , \tag{2}
\end{align*}

\begin{align*}
{f_{ \sin , i}} ( { \bf F} ) = \max \{ \min \{ {f_{ \max }} , { ( {W_{ \sin }}{ \bf F} + {{ \bf b}_{ \sin }} ) _i} \} , - {f_{ \max }} \} , \tag{3}
\end{align*}

The weight matrices *W*_cos_ and *W*_sin_ have *N* × *M* elements, where *M* is the number of mechanosensors and *N* is the number of oscillators. The bias vectors **b**_cos_ and **b**_sin_ have *N* elements. The *i*-th element of *W***F** + **b** is represented by (*W***F** + **b**)_*i*_. The functions *f*_cos,*i*_(**F**) and *f*_sin,*i*_(**F**) are clipped within [−*f*_max_, *f*_max_] to prevent obtaining very large feedback that would result in unrealistically fast rotation of phase oscillators. Let the maximum angular velocity possible in Equation (1) be *ω*_max_. The relationship between *f*_max_ and *ω*_max_ is written as follows:

\begin{align*}
{ \omega _{ \max }} = \omega + \sqrt 2 {f_{ \max }}.
\end{align*}

The constant *f*_max_ should be chosen so that a robot can follow the oscillation at *ω*_max_. In the following numerical experiments, *f*_max_ = 1 is chosen as a default value.

Various reflections can be realized by Equation (1) if the parameters are appropriately determined because the equation is equivalent to

\begin{align*}
{ \dot \phi _i} = \omega + {A_i} ( { \bf F} ) \sin ( { \phi _i} - {B_i} ( { \bf F} ) ) .
\end{align*}

This indicates that it is possible to design an oscillator that converges to an arbitrary phase with an arbitrary magnitude when it receives certain mechanosensor values.

### PGPE for controller optimization

To automatically tune feedback to the CPG-based controller, parameters *W*_cos_, *W*_sin_, **b**_cos_, and **b**_sin_ in Equations (2) and (3) are optimized using PGPE.^15^ In this study, we briefly explain the optimization procedure using PGPE. At the beginning, a vector of parameters to be optimized ***μ*** = (*μ*_1_, …, *μ_l_*)^T^ is initialized to **0**. The total amount of tunable parameters is represented by *l*. In each epoch, *K* perturbation vectors are sampled from the multidimensional Gaussian distribution

\begin{align*}
{ \bm{\epsilon} ^{ [ 1 ] }} , \; \cdots , \;{ \bm{\epsilon} ^{ [ K ] }} \sim { \cal N} ( { \bf 0} , \;{ \rm{diag}} ( \sigma _1^2 , \; \cdots , \; \sigma _l^2 ) ) .
\end{align*}

A perturbation vector ***ε***^[*k*]^ has the same element number as that of ***μ***. Standard deviations ***σ*** = (*σ*_1_, ⋯, *σ_l_*)^T^ determine the size of the perturbation. Then, 2*K* sets of parameters are generated using the perturbation vectors.

\begin{align*}
{{ { \bm{\theta} }}^{ + [ k ] }} = { { \bm{\mu} }} + {{ \bm{\epsilon}} ^{ [ k ] }} , \quad {{ { \bm{\theta} }}^{ - [ k ] }} = { { \bm{\mu} }} - {{\bm{\epsilon}} ^{ [ k ] }} \; \; \; ( 1 \le k \le K ).
\end{align*}

Each of the 2*K* parameter sets is evaluated independently by actually running a robot for one episode per parameter set. Thus, 2*K* episodes are carried out during an epoch, and 2*K* reward values are yielded. For numerical experiments conducted in this article, a reward is the displacement of the middle segment point mass along the *x*-axis during an episode, unless otherwise specified; for example, in the case of a five-segment caterpillar, this is the point mass of the third segment from the tail. The rewards are bundled into two vectors: **r**^+^ = (*r*^+[1]^, …, *r*^+[*K*]^)^T^ and **r**^−^ = (*r*^−[1]^, …, *r*^−[*K*]^)^T^. A reward for a parameter set ***θ***^+[*k*]^ is *r*^+[*k*]^ and that of ***θ***^−[*k*]^ is *r*^−[*k*]^.

Now that rewards for sampled parameter sets are obtained, we use these values to update ***μ*** and ***σ***. Since PGPE is a policy gradient-based method, ***μ*** and ***σ*** are updated so that the following reward expectation is maximized:

\begin{align*}
J ( { { \bm{\mu} }} , {{ \bm{\sigma} }} ) = \int_ \Theta { \int_h p } ( h \vert { { \bm{\theta} }} ) p ( { { \bm{\theta} }} \vert { { \bm{\mu} }} , { { \bm{\sigma} }} ) r ( h ) dhd \theta. \tag{4}
\end{align*}

The sequence of state-action pairs produced by a robot during an episode is denoted by history *h*. The expression *p*(***θ*** | ***μ***, ***σ***) represents the probability of a parameter set ***θ*** being sampled from Gaussian distribution ${ \cal N} ( { { \bm{\mu} }} , { { \bm{\sigma} }} )$, and *p*(*h* | ***θ***) represents the probability of history *h* being generated by a robot with a parameter set ***θ***. The reward assigned to a history *h* is given by *r*(*h*). Differentiating J in [Equation (4)] with regard to *μ_i_* yields


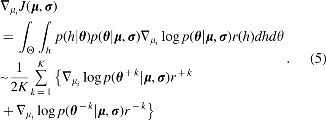


Because $\theta _i^{ \pm k} = { \mu _i} \pm \epsilon _i^k$ and $\theta _i^{ \pm k} \sim { \cal N} ( { \mu _i} , { \sigma _i} )$, the following equation holds:

\begin{align*}
{ \nabla _ { { \mu _i } } } \log p ( { { { \bm { \theta } } } ^ { \pm k } } \vert { { \bm { \mu } } } , { { \bm { \sigma } } } ) = { \frac { \theta _i^ { \pm k } - { \mu _i } } { \sigma _i^2 } } = \pm { \frac { \epsilon _i^k } { \sigma _i^2 } } . \tag { 6 } 
\end{align*}

Substituting Equation (6) into Equation (5) yields

\begin{align*}
{ \nabla _ { { \mu _i } } } J ( { { \bm { \mu } } } , { { \bm { \sigma } } } ) \sim \frac { 1 } { { 2K \sigma _i^2 } } \mathop \sum \limits_ { k = 1 } ^K { \epsilon _i^k } ( { r^ { + k } } - { r^ { - k } } )
\end{align*}

Given step size ${ \alpha _i} = \alpha 2K \sigma _i^2$ for each *μ_i_*, where *α* is a constant, the following update rule is obtained:

\begin{align*}
{ \mu _i} \leftarrow { \mu _i} + \alpha \mathop \sum \limits_{k = 1}^K { \epsilon _i^k} ( {r^{ + k}} - {r^{ - k}} ). \tag{7}
\end{align*}

The same applies to ***σ***. From the equation

\begin{align*}
{ \nabla _ { { \sigma _i } } } \log p ( { { { \bm { \theta } } } ^ { \pm k } } \vert { { \bm { \mu } } } , { { \bm { \sigma } } } ) = { \frac { { { ( \epsilon _i^k ) } ^2 } - \sigma _i^2 } { \sigma _i^3 } } ,
\end{align*}

the update rule of ***σ*** is derived:

\begin{align*}
{ \sigma _i } \leftarrow { \sigma _i } + \alpha \mathop \sum \limits_ { k = 1 } ^K { { \frac { { { ( \epsilon _i^k ) } ^2 } - \sigma _i^2 } { { \sigma _i } } } } ( { r^ { + k } } + { r^ { - k } } ). \tag { 8 } 
\end{align*}

In summary, the following operations are conducted to update ***μ*** and ***σ***:


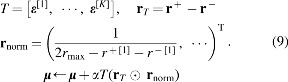



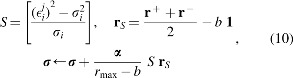


where *r*_max_ is the maximum reward obtained so far, $\epsilon _i^j$ is the *i*-th value of ***ε***^[*j*]^, and **1** is a vector of ones. The operator ⊙ denotes element-wise multiplication. The baseline *b*, which is a moving average of rewards, is defined as $b = ( 1 - \gamma ) b + \gamma \tilde r$, where *γ* is a smoothing factor and $\tilde r$ is the average of 2*K* rewards. In the following numerical experiments, *γ* = 0.01 is used as a default. Although Equations (9) and (10) are based on Equations (7) and (8), there are some alterations made for training stability. For more detail, please see the article that originally reported PGPE.^15^

#### SenseCPG-PGPE

SenseCPG-PGPE is a method to automatically explore behaviors of caterpillar-like soft-bodied robots. It consists of the components mentioned above and is illustrated in Figure 1. The CPG-based controller controls the actuators, and mechanosensor values are obtained through body–environment interactions. The feedback controller takes the mechanosensor values as input and calculates feedback to the CPG-based controller according to Equation (1). This operation cycle is repeated to run a robot. After running for several iterations, the PGPE-based optimizer evaluates the performance of the current feedback controller and updates the parameters in Equations (2) and (3). The reward function should be defined by both the mechanical system and the target functionality. The behavior of a robot is explored and optimized through this automated process.

## Mechanical System

The proposed method was validated using a simulated caterpillar-like robot. This section explains the simulation model of the caterpillar-like robot. All the variables used in the simulation model and their default values are listed in Table 1.

**Table 1.** Variables in Simulation

*Name*	*Explanation*	*Default*
*ϕ*_seg,*i*_	Phase that controls the *i*-th RTTS	—
*ϕ*_grip,*i*_	Phase that controls the *i*-th gripper	—
*ω*	Nominal angular velocity	$3.14 \;{ \rm{rad}}{ \kern 1pt} { \mkern 1mu} {{ \rm{s}}^{ - 1}}$
$\theta _{{ \rm{seg}} , i}^{{ \rm{TS}}}$	Current angle of the *i*-th TS	—
$\theta _{{ \rm{tar}} , i}^{{ \rm{TS}}}$	Target angle of the *i*-th TS	$0 \;{ \rm{rad}}$
$\theta _{{ \rm{seg}} , i}^{{ \rm{RTTS}}}$	Current angle of the *i*-th RTTS	—
$\theta _{{ \rm{tar}} , i}^{{ \rm{RTTS}}}$	Target angle of the *i*-th RTTS	—
*θ*_grip_	Gripping threshold	0.0
Θ_max_	Maximum bending angle of RTTS	1.047 rad
Θ_min_	Maximum warping angle of RTTS	−1.047 rad
*k*_TS_	Spring constant of TS	$0.007 \;{ \rm{kg}}{ \mkern 1mu} {{ \rm{m}}^2}{ \mkern 1mu} {{ \rm{s}}^{ - 2}}{ \mkern 1mu} { \rm{ra}}{{ \rm{d}}^{ - 1}}$
*c*_TS_	Damping coefficient of TS	$0.007 \;{ \rm{kg}}{ \mkern 1mu} {{ \rm{m}}^2}{ \mkern 1mu} {{ \rm{s}}^{ - 1}}{ \mkern 1mu} { \rm{ra}}{{ \rm{d}}^{ - 1}}$
*k*_RTTS_	Spring constant of RTTS	$0.07 \;{ \rm{kg}}{ \mkern 1mu} {{ \rm{m}}^2}{ \mkern 1mu} {{ \rm{s}}^{ - 2}}{ \mkern 1mu} { \rm{ra}}{{ \rm{d}}^{ - 1}}$
*k*_grip_	Spring constant of grippers	$1000.0 \;{ \rm{kg}}{ \mkern 1mu} {{ \rm{s}}^{ - 2}}$
*c*_grip_	Damping coefficient of grippers	$10.0 \;{ \rm{kg}}{ \mkern 1mu} {{ \rm{s}}^{ - 1}}$
*k*_seg_	Spring constant of intersegment springs	$300.0 \;{ \rm{kg}}{ \mkern 1mu} {{ \rm{s}}^{ - 2}}$
*c*_seg_	Intersegment damping coefficient	$10.0 \;{ \rm{kg}}{ \mkern 1mu} {{ \rm{s}}^{ - 1}}$
*x*_seg,*i*_	Segment position *i*	—
*x*_grip,*i*_	Gripping point position *i*	—
*η*_viscosity_	Viscoelastic friction coefficient	$1.0 \;{ \rm{kg}}{ \mkern 1mu} {{ \rm{s}}^{ - 2}}$
*η*_static_	Static friction coefficient	0.1
*η*_dynamic_	Dynamic friction coefficient	0.1
*r*_seg_	Radius of a segment	$0.035 \;{ \rm{m}}$
*m*_seg_	Mass of a segment	$0.003 \;{ \rm{kg}}$
*g*	Gravitational acceleration	$9.8 \;{ \rm{kg}}{ \mkern 1mu} { \rm{m}}{ \mkern 1mu} {{ \rm{s}}^{ - 2}}$

RTTS, real-time tunable torsion spring; TS, torsion spring.

### Caterpillar-like robot model

**FIG. 2.**
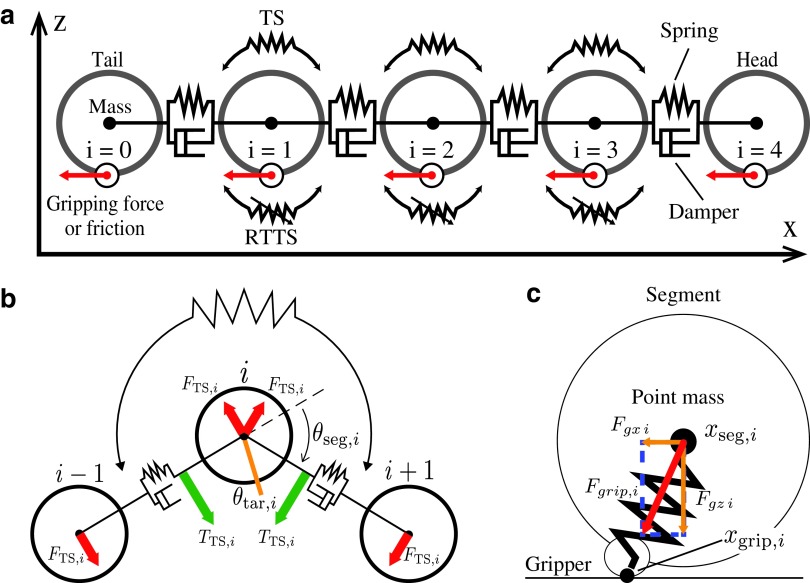
**(a)** The simulation model of a caterpillar-like robot consisting of point masses, linear springs, and dampers arranged in parallel (Kelvin–Voigt model), and two types of torsion springs: material TS and RTTS. **(b)** Model of TS and RTTS implemented on each segment. Torque *T*_TS,*i*_ is generated according to the discrepancy between the nominal angle *θ*_tar,*i*_ and the actual angle *θ*_seg,*i*_, and rotational force is applied to segments *i* − 1, *i*, *i* + 1 so that *θ*_seg,*i*_ gets closer to *θ*_tar__,*i*_. **(c)** Gripping mechanism. Gripping point *x*_grip,*i*_ is fixed when a gripper holds the ground. Spring force acts on a segment to maintain its position. RTTS, real-time tunable torsion spring; TS, torsion spring.

The robot is modeled as a system of point masses, springs, and dampers, as depicted in Figure 2a. Calculation by the simulator is based on Verlet's algorithm.^20^ One segment of a caterpillar corresponds to one point mass in the simulation. Gravity is applied to each segment which is −*m*_seg_*g* along the *z*-axis, where *m*_seg_ is the mass of each segment and *g* is the gravitational acceleration. Two adjacent segments are connected using the Kelvin–Voigt model^21^ of a spring and a damper modeling viscoelasticity. The nominal length of the spring is 2 × *r*_seg_. The value *r*_seg_ is a virtual radius of a segment. Although a segment is modeled by a point mass, we introduced this radius for convenience.

A torsion spring is embedded in every segment except for the head and tail segments. As depicted in Figure 2b, a torsion spring generates torque around it and applies force on adjacent segments. The torque corresponds to the difference between the target and actual angles.

\begin{align*}
{ T_ { { \rm { TS } } , i } } = - { k_ { { \rm { TS } } } } ( \theta _ { { \rm { seg } } , i } ^ { { \rm { TS } } } - \theta _ { { \rm { tar } } , i } ^ { { \rm { TS } } } ) - { c_ { { \rm { TS } } } } { \frac { d \theta _ { { \rm { seg } } , i } ^ { { \rm { TS } } } } { dt } } .
\end{align*}

The force strength applied on an adjacent segment is

\begin{align*}
{ F_ { { \rm { TS } } , i } } = { \frac { { T_ { { \rm { TS } } , i } } } { 2 { r_ { { \rm { seg } } } } } } .
\end{align*}

The target angles of torsion springs are set to $\theta _{{ \rm{tar}} , i}^{{ \rm{TS}}} = 0$, so the segments align in parallel by default. For simplicity, the length between two adjacent segments is regarded as fixed to 2*r*_seg_ when calculating force.

Apart from the normal torsion springs mentioned above, a controllable torsion spring called a real-time tunable torsion spring (RTTS) is embedded in every segment except for the head and tail. An RTTS models muscle and its target angle $\theta _{{ \rm{tar}} , i}^{{ \rm{RTTS}}}$ are controlled by an oscillator phase *ϕ*_seg,*i*_. Note that a target angle is positive when bending (i.e., under a straight line) and is negative when warping (i.e., above a straight line). A target angle is controlled as follows:

\begin{align*}
\theta _ { { \rm { tar } } , i } ^ { { \rm { RTTS } } } = \frac { { { \Theta _ { \max } } - { \Theta _ { \min } } } } { 2 } ( 1 - \cos { \phi _ { { \rm { seg } } , i } } ) + { \Theta _ { \min } } , \tag { 11 } 
\end{align*}

where Θ_max_ is the maximum angle of bending, and Θ_min_ is the maximum angle of warping, which is negative. Damping force is not included in the RTTS.

Each segment has a gripper. Once a gripper holds the substrate, the gripping point *x*_grip,*i*_ is fixed and does not move. During gripping, the force along the *x*-axis,

\begin{align*}
{ F_ { { \rm { grip } } , i } } = - { k_ { { \rm { grip } } } } ( { x_ { { \rm { seg } } , i } } - { x_ { { \rm { grip } } , i } } ) - { c_ { { \rm { grip } } } } { \frac { d { x_ { { \rm { seg } } , i } } } { dt } } ,
\end{align*}

acts on a segment. The coefficient *k*_grip_ is set to a large value to model a hard spring. The force along the *z*-axis on a segment is cancelled during gripping. In the following numerical experiments, a gripper is controlled by an oscillator because the caterpillar robot is given a task to climb a step and walk on the ceiling. Thus, we judged it to be appropriate to control a gripper independently and assigned a dedicated oscillator. It holds the substrate if sin *ϕ*_grip,*i*_ ≤ *θ*_grip_ and a segment is on the substrate.

A frictional force acts on a segment while it is on the substrate but not gripping it. The force consists of viscosity friction ${ \eta _{{ \rm{viscosity}}}}{ \dot x_i}$ and either static friction min{*F*_seg,*i*_, *η*_static_*F*_N_} or dynamic friction *η*_dynamic_*F*_N_, where *F*_seg,*i*_ is the resultant force applied on the *i*-th segment (friction not included) and *F*_N_ is the normal force from the substrate.

### Mechanosensor values

For mechanosensors, the torque generated by RTTSs and the *x* and *z* components of tensions between grippers and segments were used. These values were chosen because we considered them easy to obtain using off the shelf sensors. The torque generated by an RTTS can be directly estimated from the discrepancy between the target angle and current angle of the actuator. The *x* and *z* components of tension between a gripper and a segment can be measured by observing the deformation of the gripper along the *x*-axis and the distance between a segment and the ground, respectively, using a photo reflector, as was done by Umedachi *et al*.^11^

Although there are additional state values, such as tension generated by a spring between two segments that models material elasticity, these values were not used because methods to measure such values were unavailable. However, from the perspective of biology, real caterpillars most likely sense such values. Thus, in future work, we will investigate methods to sense such values and explore their effect on soft robot behaviors.

In summary, the state input **F** for a five-segment caterpillar, which was used in most experiments, has the following 13 dimensions:

\begin{align*}
{ \bf F} = { \left( {{T_{s1}} , {T_{s2}} , {T_{s3}} , {F_{gx0}} , \cdots , {F_{gx4}} , {F_{gz0}} , \cdots , {F_{gz4}}} \right) ^{ \rm{T}}} , \tag{12}
\end{align*}

where *T_si_* represents torque generated by an actuator on the *i*-th segment, and *F_gxj_* and *F_gzj_* are *x* and *z* components of tension between the *j*-th segment and a corresponding gripper, respectively.

## Experiments

Several numerical experiments were conducted to study the effectiveness of the proposed method. The proposed method was compared to two baseline alternatives to consider the importance of mechanosensory feedback.

1. LocalSenseCPG-PGPE: In this approach, an oscillator only monitors mechanosensors that are close to the actuator it controls. An RTTS on the *i*-th segment is controlled using the magnitude of torque generated by itself and tension on the grippers on the *i*-th and adjacent segments. A gripper on the *i*-th segment is controlled using the magnitude of torque generated by an RTTS on the *i*-th segment and the tension applied to itself. The parameters are optimized using PGPE.^15^
2. Kuramoto-PGPE: Oscillators are ruled by Kuramoto's model,^22,23^ which is shown in Equation (13). The number of oscillators is *N*. An oscillator is influenced by all other oscillators, and the magnitude of influence is *κ_ij_*. *κ_ij_* and the target phase difference *ρ_ij_* are optimized using PGPE. No mechanosensors are used in this method.

\begin{align*}
{ \dot \phi _i} = \omega + \mathop \sum \limits_{j = 0}^N {{ \kappa _{ij}}} \sin ( { \phi _i} - { \phi _j} - { \rho _{ij}} ) . \tag{13}
\end{align*}

### Comparison of training methods

This section compares the training performance of the proposed method to other baseline approaches. The following three other reinforcement learning methods were used as baselines: deep deterministic policy gradient (DDPG),^24^ trust region policy optimization (TRPO),^25^ and proximal policy optimization (PPO).^26^ These are popular reinforcement learning methods for a continuous action space. The proposed method is simultaneously compared to its alternatives, namely, LocalSenseCPG-PGPE and Kuramoto-PGPE, which have different sensor–actuator coordinations.

In the experiment, linear functions that calculate *f*_cos,*i*_ and *f*_sin,*i*_ in Equation (1) were optimized using each reinforcement learning method. A five-segment caterpillar was used. All controllers were trained for 500 epochs. For DDPG, TRPO, and PPO, one epoch was locomotion over a 50 s duration. A caterpillar was placed at *x* = 0 at the beginning of an epoch, ran for 50 s, and was then reset and placed at *x* = 0 again. Since the state update was conducted every 0.01 s, there were 5000 updates during an epoch. In DDPG, TRPO, and PPO, reward discount *γ* was set to 0.99, and Adam^27^ was used for optimization. The conditions for each method were as follows.

#### Deep deterministic policy gradient

A simple linear transform bounded by tanh was used for the policy, that is, tanh(*W***F** + b), where *W* and b are a weight matrix and a bias, respectively. For an action–value function, a perceptron with one hidden layer was used. A state vector with 13 dimensions was fed to the input layer of the network. Outputs from the input layer (7 dimensions) and an action (16 dimensions) were concatenated and fed to the hidden layer. The activation function in the network was tanh. The Ornstein–Uhlenbeck process with mean *μ* = 0.0, noise scale *σ* = 0.4, and growth rate *θ* = 0.15 was adopted for exploration noise. The capacity of the experience replay buffer was 1 × 10^5^, and the parameter update started when 1 × 10^4^ experiences (i.e., pairs of state, action, and reward) were stored.

#### Trust region policy optimization and proximal policy optimization

The policy network structure of DDPG was used. A perceptron with one hidden layer of 7 units was used for a value function. Gaussian noise with mean *μ* = 0.0 was added to action during training for exploration noise. The variance of the noise was adjusted automatically.

#### Policy gradients with parameter-based exploration

One epoch consisted of 20 independent episodes, and one episode lasted 50 s. The initial standard deviations of Gaussian distribution used in PGPE were set to *σ* = 2.0. SenseCPG-PGPE, LocalSenseCPG-PGPE, and Kuramoto-PGPE were trained in the same scheme using PGPE.

**FIG. 3.**
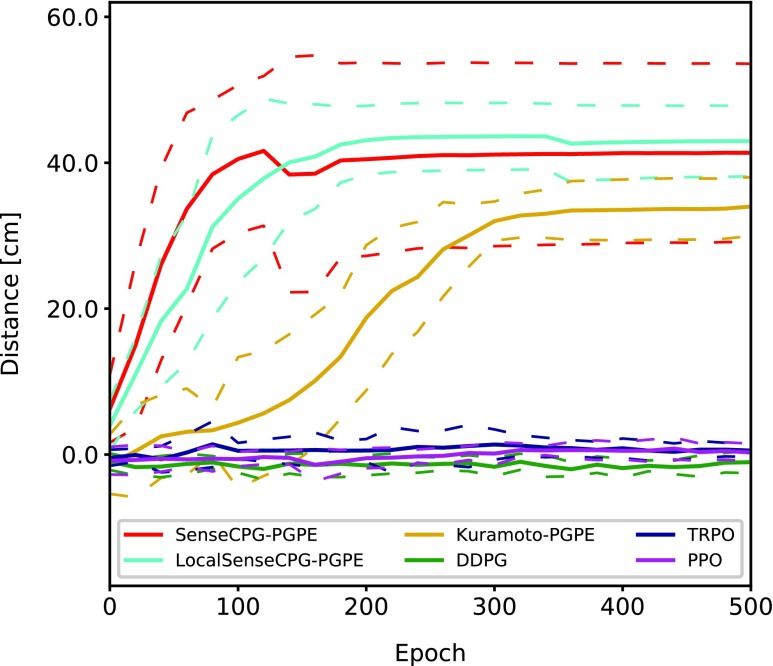
Learning curves of several reinforcement learning methods and mechanosensory feedback compositions. Each solid curve is an average of over 10 training trials, and the *dotted lines* show standard deviation. Curves are smoothed over every 20 epochs. DDPG, TRPO, and PPO, which update the policy at every step, yielded poor performance compared to PGPE. DDPG, deep deterministic policy gradient; PPO, proximal policy optimization; TRPO, trust region policy optimization.

Figure 3 shows the averaged learning curves over 10 trials. PPO, DDPG, and TRPO yielded poor results compared to PGPE. Among the three methods trained using PGPE, Kuramoto-PGPE, which does not use any mechanosensory feedback at all, yielded the poorest performance, which implies that mechanosensory feedback is useful even for monotonous periodical behavior. The average gait frequencies of SenseCPG-PGPE, LocalSenseCPG, and Kuramoto were 0.521 (±0.082), 0.543 (±0.122), and 0.627 (±0.068) Hz, respectively.

### Controlling many segments

**FIG. 4.**
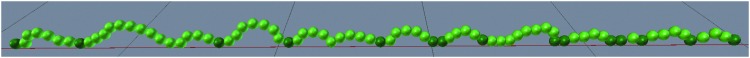
Snapshot of a caterpillar with 80 segments.

Controllers for caterpillars with many segments (40 and 80 segments) were trained in this experiment. There are 118 sensor inputs for a caterpillar with 40 segments, comprising vertical and horizontal force sensors for 40 grippers and 38 tension sensors on RTTSs along the body. Feedback for 40 grippers and 38 RTTSs must be calculated at every step. Thus, there are 118 × 78 = 9204 sensor–actuator compositions to be considered. In the case of a caterpillar with 80 segments, 238 × 158 = 37,604 compositions must be considered. Figure 4 is a snapshot of the 80-segment caterpillar. A controller was trained for 2000 epochs using PGPE.

**FIG. 5.**
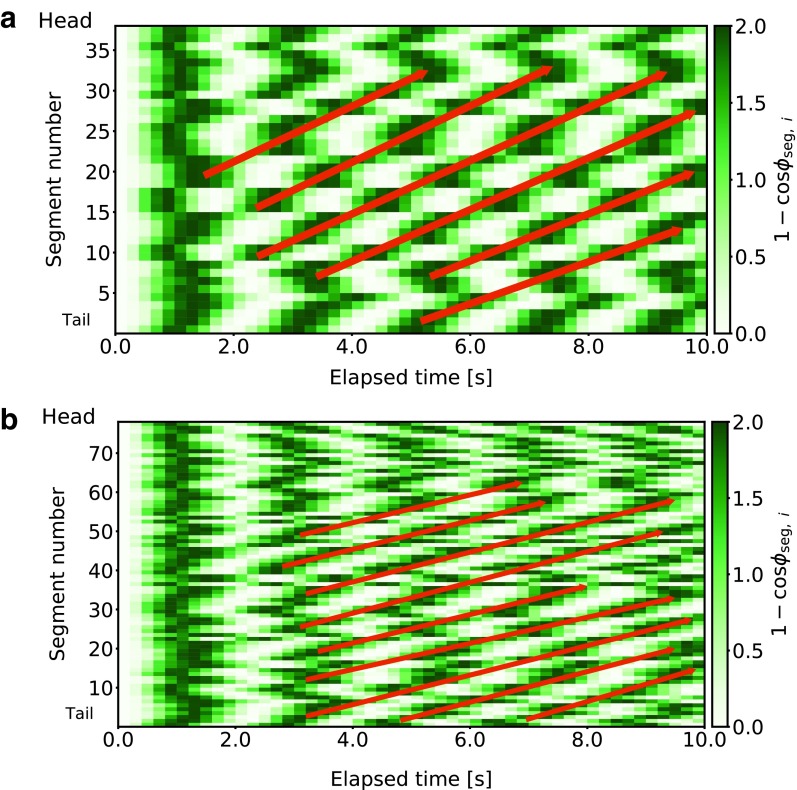
Time development of the phases of oscillators that control RTTSs in a caterpillar with **(a)** 40 segments and **(b)** 80 segments. Value of 1 −cos(*ϕ_seg_*_,*i*_) is visualized using different chroma of *green*. The oscillators were in phase at the beginning, but a diagonal alignment of *dark green* emerged.

The obtained controller drove the 40-segment caterpillar 8.13 m in 100 s. The frequency of an oscillator was ∼0.485 Hz. Since the length of the caterpillar was 2.8 m, it proceeded 290% of its body length during the period. Figure 5a visualizes the time development of the phases in oscillators for RTTSs, that is, the time development of 1 − cos *ϕ*_seg,*i*_. The value 1 − cos *ϕ*_seg,*i*_ was used because it is directly related to the target angle of a segment actuator, as in Equation (11). Dark green (i.e., 1 − cos *ϕ*_seg,*i*_ being 2.0) and white (i.e., 1 − cos *ϕ*_seg,*i*_ being 0.0) correspond to the target angle of an actuator being at its maximum (i.e., bending) and minimum (i.e., warping), respectively. At the beginning of an episode, all the oscillators were in phase. The diagonal alignment of dark green appeared as time elapsed, which indicates that bending command flowed from the tail to the head. The figure also shows that bending did not occur one segment after another. Instead, a group of segments bent at once, and this group excitation flowed. Figure 5b shows the time development of the phases in the 80-segment caterpillar. The same trend was observed here. To see the flow of bending from the tail to the head, we refer readers to the Supplementary Video S1.

We also compared displacement per body length during a gait cycle of 5-, 40-, and 80-segment caterpillars. The trained five-segment caterpillar showed inching behavior with a gait frequency of 0.446 Hz, and it moved 4.67 m during one episode (100 s). Thus, its averaged displacement was ${ \frac { 4.67 } { 100 } } \times \frac { 1 } { { 0.446 } } \fallingdotseq \ 0.105 { \mkern 1mu } { \kern 1pt } { \kern 1pt } { \rm { m } }$ during one cycle, which is 0.105 / (5 × 0.07) ≒ 0.300 body length. In contrast, displacement per body length during a cycle of the 40-segment caterpillar was 0.0599 and that of the 80-segment caterpillar was 0.00257. The five-segment caterpillar moved the largest distance per body length during a cycle because it used a gait pattern called inching. However, the actuators cannot support the longer body to realize this gait pattern, so a gait pattern called crawling emerged in the 40-segment caterpillar. As we will discuss later, for a given body length, crawling is slower than inching. This explains why the displacement per body length during a cycle of the 40-segment caterpillar was smaller compared with the 5-segment caterpillar. The 80-segment caterpillar achieved the smallest displacement per body length during a cycle; this is likely due to training time being insufficient. We stopped the training of this caterpillar before convergence because its simulation was too time consuming. Conducting more extended training with an improved simulator may help to improve the policy.

### Nonsteady motion

The controller was trained to climb a step in a path to clarify whether the CPG-based controller can learn to perform a task that requires nonsteady motion using mechanosensors. A step of height 8.0 cm, which is higher than a segment diameter was placed at *x* = 35 cm. To judge whether a segment is being hampered by the step, the simulator checks the following condition every step:

\begin{align*}
{z_{{ \rm{seg}} , i}} \le h ( {x_{{ \rm{seg}} , i}} ) + {r_{{ \rm{seg}}}} - { \epsilon _{{ \rm{hampered}}}} , \tag{14}
\end{align*}

where *z*_seg,*i*_ and *x*_seg,*i*_ denote the vertical and horizontal position of a segment, respectively. The height of the path at position *x*_seg,*i*_ is denoted by *h*(*x*_seg,*i*_). The small constant *ε*_hampered_ was used to distinguish the situation from the normal landing of a segment, and it was set to 1.0 × 10^−3^. If the condition [Inequality (14)] is satisfied, the segment is judged as being hampered and cannot move forward in the simulation. A sensor that monitors whether the head segment is hampered was added, and its output was fed to all oscillators; more specifically, a variable that is equal to 1 if the head segment is hampered and equal to 0 if not was prepared. This value was concatenated with other sensor values and fed into *f*_cos,*i*_ and *f*_sin,*i*_ in Equation (1). The weights for the value were optimized by PGPE in the same way as the other weights. A five-segment caterpillar was used, and the controller was trained for 400 epochs using PGPE.

**FIG. 6.**
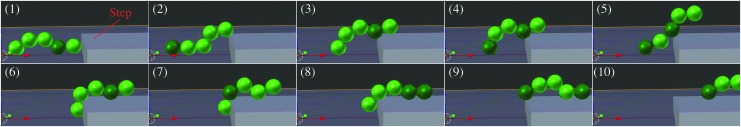
Snapshots of climbing a step. The step was located at *x* = 35 cm and its height was 8.0 cm, which is higher than the diameter of a segment. The *dark green color* indicates that a segment is gripping the substrate.

**FIG. 7.**
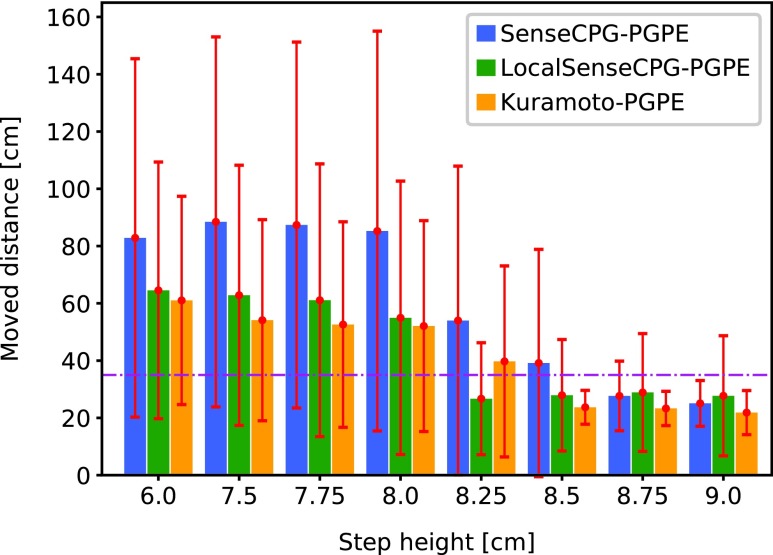
Comparison of locomotion distance in 100 s, during which time the robot must climb one step of a different height. A step was placed at *x* = 35 cm. The controllers were trained to navigate a step of height 8 cm. The *horizontal dashed line* shows the position of a step. Each *bar* presents the average of 10 separately trained controllers. The error bars are large due to some training trials failing, even with the proposed SenseCPG-PGPE. The robot using Kuramoto-PGPE barely managed to climb a step, whereas the robot using the proposed method was able to climb an average highest step of 8.5 cm.

Figure 6 shows how the caterpillar managed to climb the step. When the hamper sensor on the head detected the step, it raised the front segments and put the head segment on the step. Then, the segments were transported to the top of the step one by one. Although a CPG-based controller is mostly used for periodical steady motion, a reactive motion was achieved here. Figure 7 shows the climbing performance on steps of different heights. All controllers here were trained on the step of 8.0 cm height. SenseCPG-PGPE, LocalSenseCPG-PGPE, and Kuramoto-PGPE were compared. The averaged gait frequencies were 0.513 (±0.055), 0.476 (±0.143), and 0.622 (±0.207) Hz, respectively. Until a height of 8.5 cm, the proposed controller achieved the fastest locomotion. As the height increases, climbing becomes more difficult, and even controllers trained with the proposed method could not successfully climb the step. A different policy is most likely required to climb a higher step.

### Different body dynamics

Controllers were trained under two different body configurations (A and B). In condition A, the RTTSs and springs between adjacent segments were weakened, that is, their spring constants were set to *k*_RTTS_ = 0.7 and *k*_seg_ = 20.0, respectively. In condition B, the RTTSs and springs were made stiffer, that is, their spring constants were set to *k*_RTTS_ = 3.0 and *k*_seg_ = 300.0, respectively. For the latter condition, an RTTS can lift the whole body, which weighs 150 g. In addition, the bending angle was increased to 1.570 in condition B, whereas both bending and warping angles were set to 1.047 in condition A. As a result, bending was easier for the caterpillar in condition B. A five-segment caterpillar was used, and controllers were trained for 200 epochs with PGPE.

**FIG. 8.**
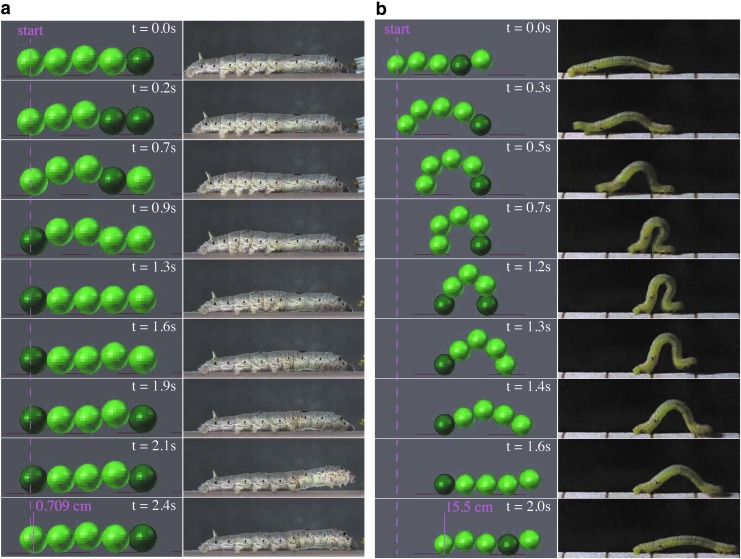
Snapshots of different gait patterns obtained for different body configurations. The *dark green color* indicates that a segment is gripping the substrate. **(a)** Crawling acquired under condition A (*left*), where the actuator was relatively weak. A direct wave was observed, as in the crawling of silkworms (*right*). **(b)** Inching acquired under condition B, where the actuator was ∼4.3 times stronger than in condition A. The segments were able to bend from −1.407 to 1.570, and the springs between the segments were made stiffer. The *rear* and the *front ends* move in turn, resulting in limping behavior, as seen in inchworms (*right*).

In condition A, crawling was observed after optimization, as shown on the left side of Figure 8a. The gait frequency was 0.437 Hz, and displacement during one episode (100 s) was 0.298 m. A cycle starts by lifting the rear segment, and the lifting motion propagates toward the front. Crawling is adopted in relatively large caterpillars such as silkworms, as is shown on the right side of Figure 8a. In condition B, on the other hand, a distinctive gait pattern called inching was observed, as is shown in the left side of Figure 8b. The gait frequency was 0.503 Hz, and displacement during one episode (100 s) was 7.668 m. In this pattern, the head segment holds the substrate first, and the tail segment is dragged forwards. Then, the tail segment holds the substrate, and the body extends to move the head segment forward. This gait pattern is adopted in inchworms, as is shown on the right side in Figure 8b. Since the RTTSs were strong enough to lift the middle three segments, inching was possible in condition B. However, the gait pattern converged to crawling in condition A, which is likely because the RTTSs did not provide sufficient power.

### Different environmental conditions

Controllers were trained under different gravity directions. In the first condition, gravity acted to keep the body on the substrate, as in normal locomotion on the ground. In the second condition, gravity acted to pull the body away from the substrate, as in locomotion on the ceiling. A penalty was imposed when the body left the substrate, that is, when none of the segments was on the substrate, to make the controller learn a policy that keeps the body on the substrate. Hence, the following reward was adopted:


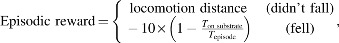


where ${T_{{ \rm{on \ substrate}}}}$ is the duration before falling off and ${T_{{ \rm{episode}}}}$ is an episode duration. Initial phases of oscillators for grippers were set to $\frac { 3 } { 2 } \pi$ instead of the default value 0 to prevent the body from falling off the ceiling at the beginning of an episode. This phase adjustment made the caterpillar start an episode in the gripping state. The default gripping duration was extended to accelerate learning, that is, the gripping threshold *θ*_grip_ was changed from 0 to $\sin \frac { \pi } { 4 }$. A five-segment caterpillar with the following configurations were used: *k*_seg_ = 300.0, *c*_seg_ = 10.0, and *k*_RTTS_ = 5.0. Controllers were trained for 200 epochs with PGPE in both conditions.

**FIG. 9.**
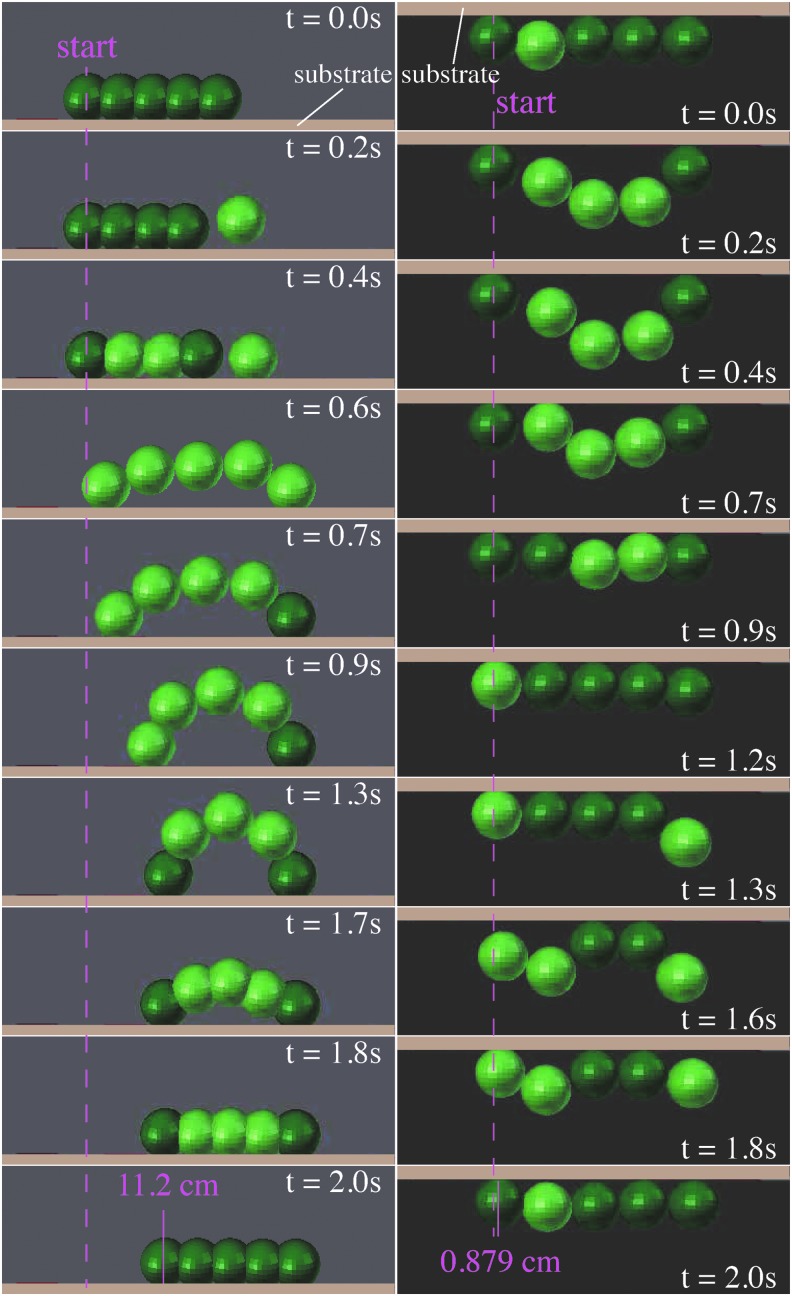
Snapshots of gait patterns acquired for different gravity settings: locomotion on the ground (*left column*) and locomotion on the ceiling (*right column*). The *brown area* is the substrate. Segments in *dark green* are gripping the substrate in each frame. **(a)** Inching acquired for locomotion on the ground. **(b)** Gait pattern for locomotion on the ceiling.

Figure 9a shows locomotion acquired on the ground, which is inching. The gait frequency was 0.714 Hz, and displacement during one episode (100 s) was 5.32 m. On the ceiling, however, a distinct gait pattern was observed, as shown in Figure 9b. The gait frequency was 0.508 Hz, and displacement during one episode (100 s) was 0.352 m. The end and middle segments held the substrate in turns.

## Discussion

### Partially observable Markov decision process during training

**FIG. 10.**
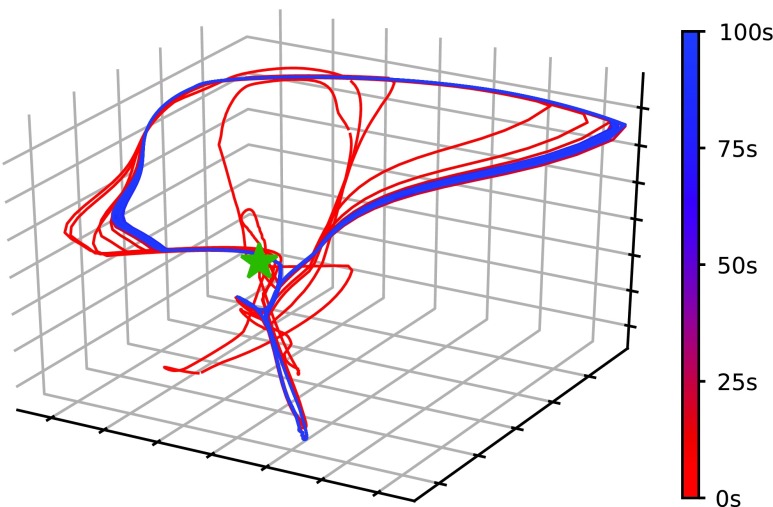
Time development of a body state. Seven values (four distances of adjacent segments and three bending angles) were standardized and reduced to three dimensions using principal component analysis. The dimension-reduced states were plotted, starting at the *green star marker*. The color of the line transitions from *red* to *blue* as time proceeds. By the time the color of the line becomes *blue*, the trajectory converged to a stationary orbit; in other words, a limit cycle was formed.

Although the system followed a partially observable Markov decision process (POMDP), PGPE, which assumes a Markov decision process, was able to learn the appropriate policy because the complexity of motion was reduced by entrainment of the oscillators. Each segment in the simulated caterpillar-like robot has two degrees of freedom, namely, bending of the body and elasticity along the direction of the body's axis. The latter is neither controllable nor observable. Usually, this causes perceptual aliasing, and countermeasures such as estimation of the real state from action history are required. However, the motion of the body converged to a stationary cycle after entrainment of oscillators, as depicted in Figure 10. Figure 10 shows the time development of the body states, that is, the bending angle of each segment and the distance between every two adjacent segments. The dimension of a body state vector, which was seven originally, was reduced to three using principal component analysis. The body state vectors were then plotted. The line gradually changes from red to blue over time, starting from the green star marker. Convergence of the orbit to a limit cycle was observed. This result implies that entrainment reduces the complexity of the motion. This is why PGPE can learn a policy well even under the POMDP.

DDPG, TRPO, and PPO failed because they could not process the temporal changes in the system dynamics. As Figure 10 shows, it takes some time for the entrainment to take place and to converge to a steady state. The three reinforcement learning methods could not find a good policy because they conduct step-based policy updates, so they try to train a single policy under different dynamics. PGPE, on the other hand, updates the policy based on the performance throughout an episode. Therefore, it learns the policy that maximizes performance after entrainment, resulting in stable locomotion.

### Phase difference modulation

Mechanosensory feedback is useful even for locomotion on flat terrain. Figure 3 shows that the performances of SenseCPG-PGPE and LocalSenseCPG-PGPE were better compared with Kuramoto-PGPE, although some trials of SenseCPG-PGPE showed poor performance due to significant variance in training. The result is unintuitive because it is locomotion on flat terrain, and as long as parameters are optimized, performance seems to converge to the same level, regardless of sensor information incorporation. As reported in Comparison of Training Methods section, the frequency of Kuramoto-PGPE is higher compared with SenseCPG-PGPE and LocalSenseCPG-PGPE. Thus, operation frequency does not explain the lower locomotion speed of Kuramoto-PGPE.

One possible reason is that SenseCPG-PGPE and LocalSenseCPG-PGPE can modulate phase differences but Kuramoto-PGPE cannot. Figure 15 shows phase differences of oscillators for segment actuators during the step climbing experiment in Nonsteady Motion section. During the period of locomotion on the step (after *s*_0_ line), fine modulation of phase differences can be observed in SenseCPG-PGPE, whereas phase differences are constant in Kuramoto-PGPE. This fine modulation of phase differences according to body state seems to be important for faster locomotion. If this is the case, it implies that using sensor information to estimate current body state is beneficial even for monotonous periodical behaviors. Furthermore, if direct connections among oscillators are dominant, modulation is unlikely to occur. Therefore, an orchestration of oscillators through physical communication may be useful.

### Benefit of global mechanosensory feedback

**FIG. 11.**
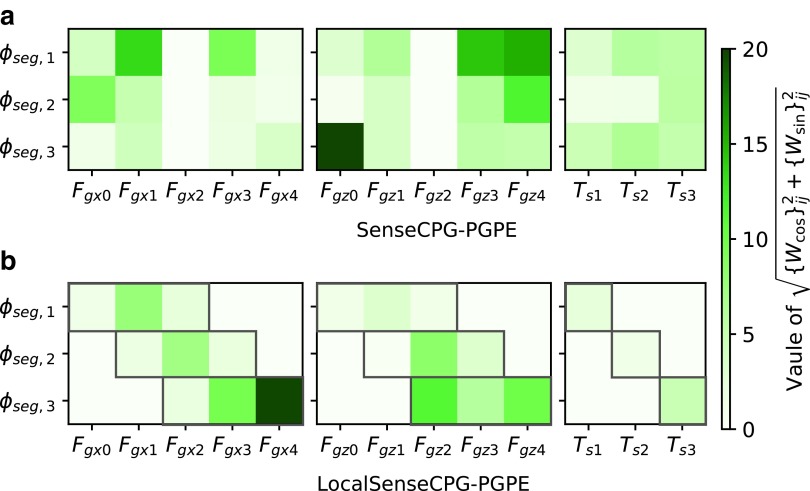
Magnitudes of feedback weights obtained for locomotion on flat terrain using **(a)** SenseCPG-PGPE and **(b)** LocalSenseCPG-PGPE. Feedback weights to oscillators for segment actuators are shown. The symbols of sensors correspond with Equation (12). For simplicity, two weights {*W*_cos_}_*ij*_ and {*W*_sin_}_*ij*_ (*i* and *j* are oscillator id and sensor id, respectively) are bundled into $\sqrt { \{ {W_{ \cos }} \} _{ij}^2 + \{ {W_{ \sin }} \} _{ij}^2}$. Weights for inactive sensors (i.e., sensors that returned zero during stable locomotion) are shown as zero. The original values of the weights are published online (Supplementary Data). In LocalSenseCPG-PGPE, only weights surrounded by the *red frames* were active.

Global mechanosensory feedback is more important for achieving complex behaviors. As Figure 3 shows, the benefit of SenseCPG-PGPE is not clear in locomotion on flat terrain, although global mechanosensory feedback was obtained in SenceCPG-PGPE as is shown in Figure 11. Worth noting is that the best controller of SenseCPG-PGPE achieved better performance than the best of LocalSenseCPG-PGPE. Thus, global mechanosensory feedback may be just as useful as local mechanosensory feedback in flat terrain locomotion. Locomotion on flat terrain might be a simple enough task such that local mechanosensory feedback alone was enough to achieve good performance.

**FIG. 12.**
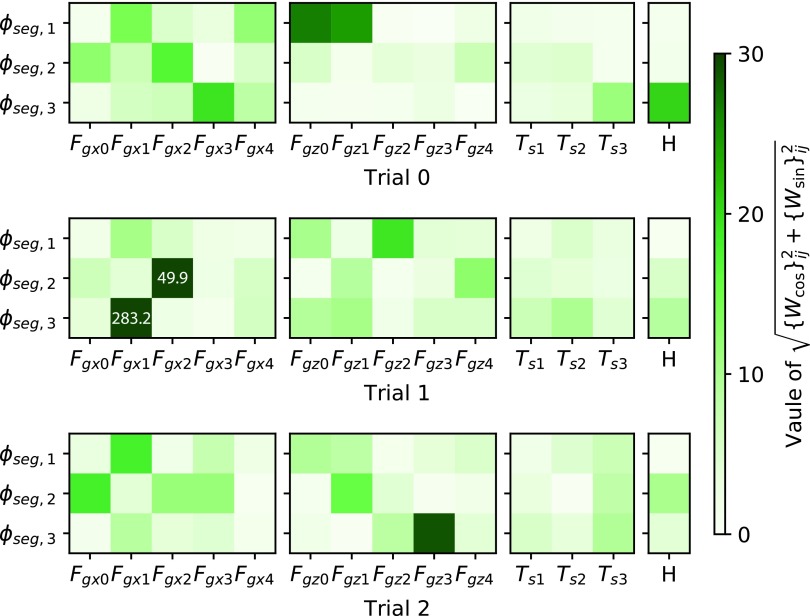
Magnitudes of feedback weights (i.e., $\sqrt { \{ {W_{ \cos }} \} _{ij}^2 + \{ {W_{ \sin }} \} _{ij}^2}$) in three best SenseCPG-PGPE controllers obtained in Nonsteady Motion section. *H* represents the sensor on the head to detect an obstacle in front. Other symbols of sensors correspond with Equation (12). The *white numbers* in the heat maps show the actual magnitudes of weights that exceed the range of the *color bar*. The actual values of the weights are published online (Supplementary Data). Caterpillars in the three trials showed similar climbing behaviors, but details differed (e.g., timing of placing each segment on the step).

In contrast, the benefit of global mechanosensory feedback is obvious when climbing a step, as shown in Figure 7. Figure 12 visualizes weight parameters of the best three SenseCPG-PGPE controllers. Strong attention to nonadjacent sensors was observed. The result makes sense because climbing a step requires the cooperation of distant body parts (e.g., pulling by the head and pushing by the tail must occur at corresponding times). Therefore, incorporating global sensory feedback is beneficial to achieve complex and tactical behaviors.

### Weights on different dynamics and environments

**FIG. 13.**
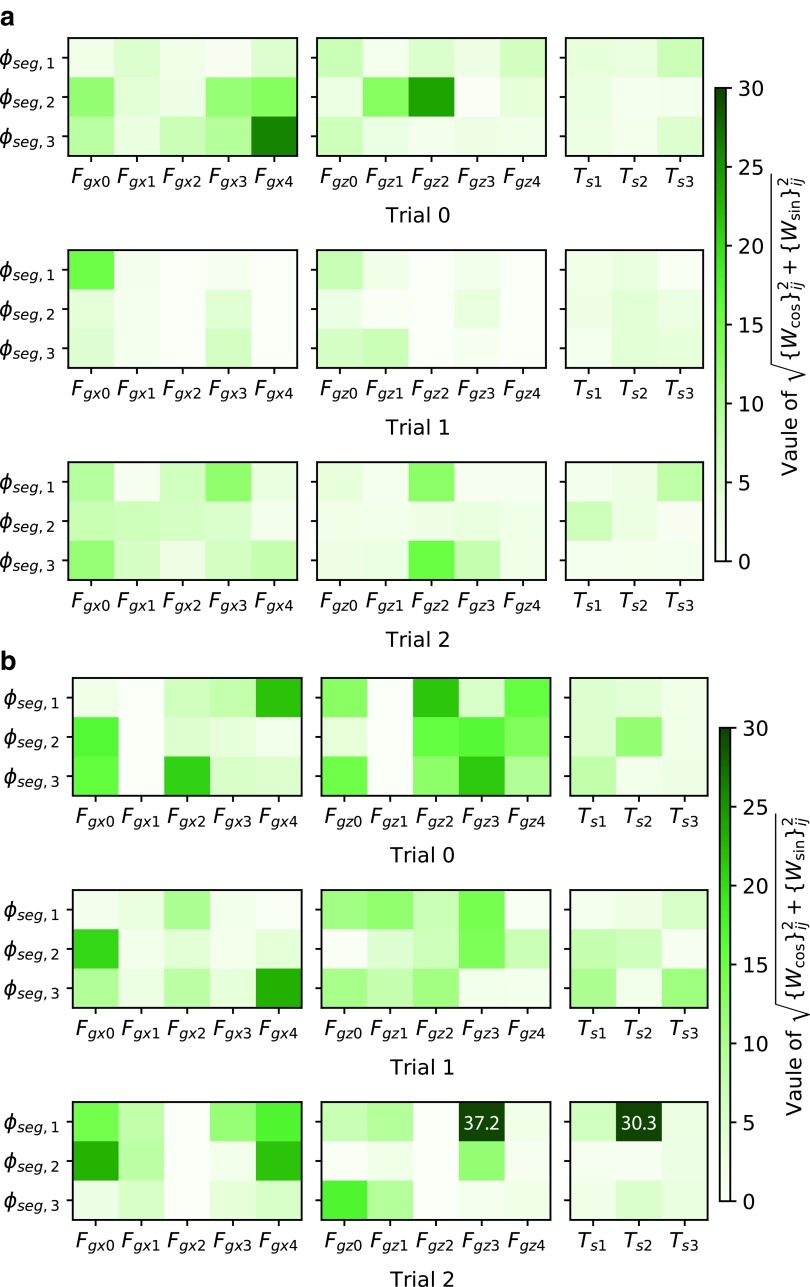
Magnitudes of feedback weights (i.e., $\sqrt { \{ {W_{ \cos }} \} _{ij}^2 + \{ {W_{ \sin }} \} _{ij}^2}$) in the best three controllers for **(a)** crawling and **(b)** inching obtained in Different Body Dynamics section. The symbols of sensors correspond with Equation (12). Weights for inactive sensors (i.e., sensors that returned zero during stable locomotion) are shown as zero. The *white numbers* in the heat maps show the actual magnitudes of weights that exceed the range of the *color bar*. The original values of the weights are published online (Supplementary Data).

Training on different body dynamics leads to different feedback mechanisms, which generate different behaviors. Figure 13 shows weights of some controllers that lead to crawling and inching. Weights for crawling are stronger than those for inching. This result is intuitive. To generate inching behavior, a strong synchronization of bending actuators is required. Hence, feedback to controller oscillators most likely needs to be more intense, and larger weights were acquired for this purpose. In contrast, such synchronization does not occur in crawling, so smaller weights were obtained. In general, however, the weight patterns seem to be different from each other, even among controllers that generated the same behavior. This implies that the solution space of feedback weights which lead to a certain behavior diverges, possibly because there was redundancy in sensor information. Thus, the correlation between a weight pattern and a resultant behavior is not clear. This is why we should utilize machine learning techniques to explore behaviors.

**FIG. 14.**
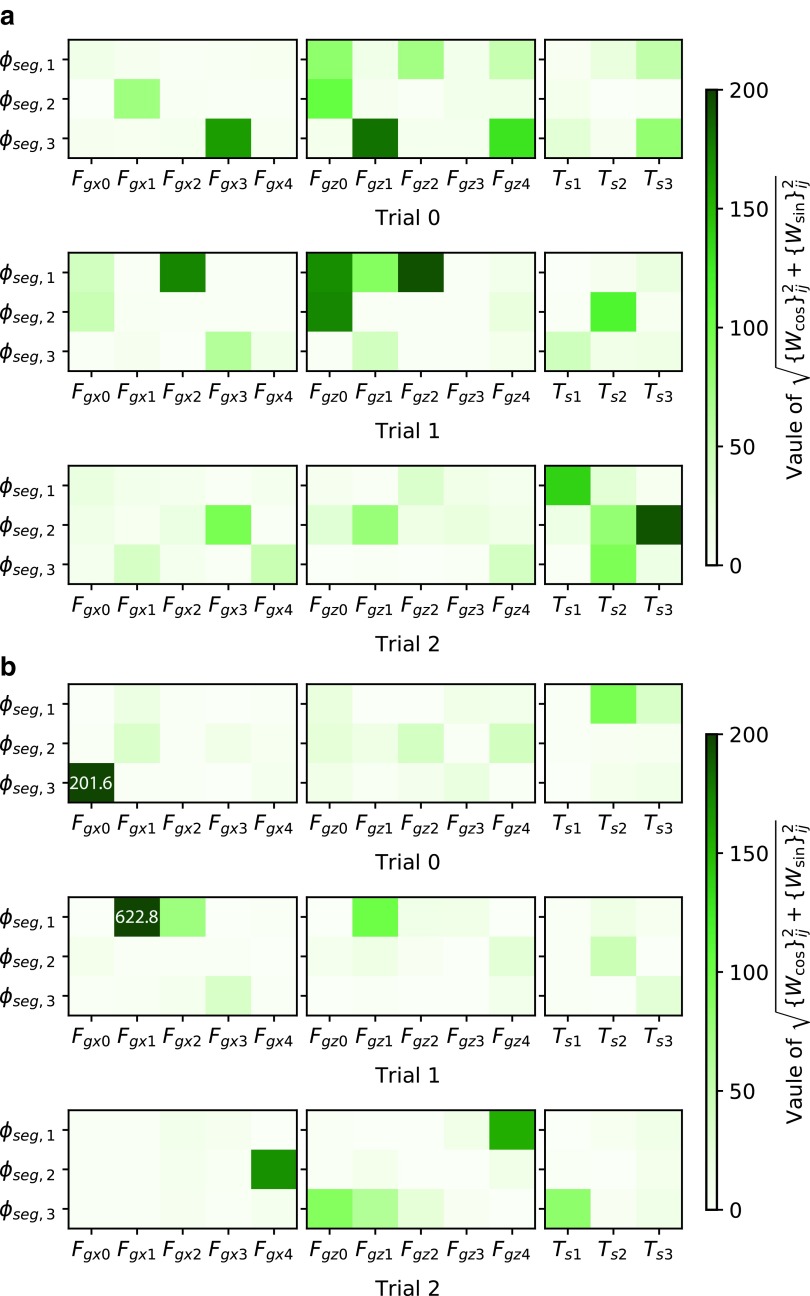
Magnitudes of feedback weights (i.e., $\sqrt { \{ {W_{ \cos }} \} _{ij}^2 + \{ {W_{ \sin }} \} _{ij}^2}$) in the best three controllers for locomotion **(a)** on the ground and **(b)** on the ceiling obtained in Different Environmental Conditions section. The symbols of sensors correspond with Equation (12). The *white numbers* in the heat maps show the actual magnitudes of weights that exceed the range of the *color bar*. Note that the weight values in **(b)** are 0.1 time smaller than the original values acquired. The original values of the weights are published online (Supplementary Data). In controllers for locomotion on the ceiling, each oscillator tends to pay keen attention to sensors on a specific gripper.

The different environments also lead to different feedback weight patterns. Figure 14 visualizes feedback weights obtained for locomotion on the ground and on the ceiling. Note that the original weights for the ceiling were ten times greater than those shown in Figure 14b. However, running the caterpillar simulation on the ceiling with controller parameters being multiplied by 0.1 did not cause performance degradation. Thus, the original values were multiplied by 0.1 for comparison of weights between environments. The unnecessarily large weights were likely due to the training process or reward function. Compared to weights in Figure 14a, weights in Figure 14b are very sparse and strong attention is limited to one or two sensors for each oscillator. For example, *ϕ*_seg,3_ of Trial 0 in Figure 14b only pays strong attention to *F*_gx0_. Such a feedback pattern appeared to prevent the body from falling off the ceiling. The resulting weight patterns may imply that each body part should pay more attention to a specific gripper so that it does not disturb the lifeline gripper, which would prevent falling.

### Behavior switching according to mechanosensory feedback

**FIG. 15.**
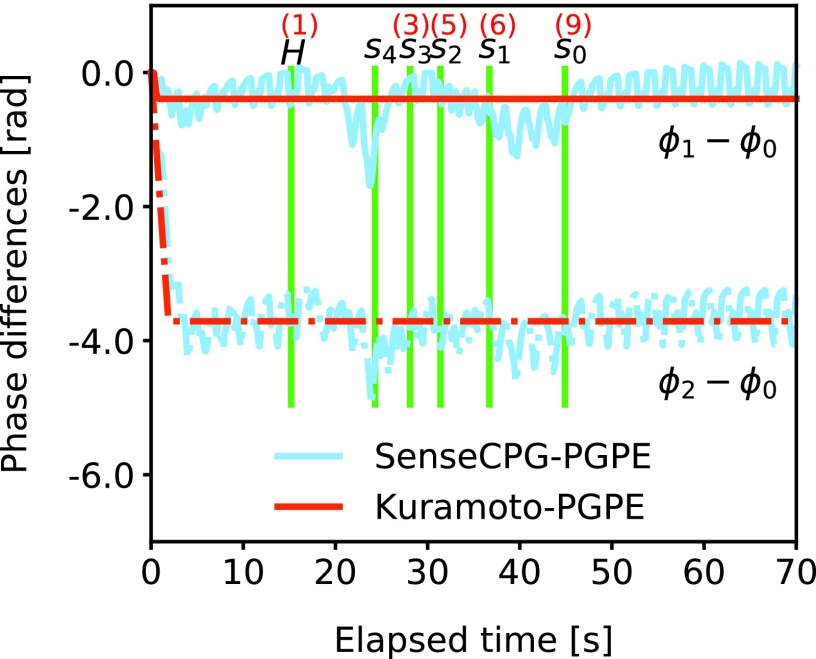
Time development of phase differences of oscillators that control RTTSs during locomotion and climbing a step. The *green line* under the *h* mark shows when the head segment first touched the side of the step. Other lines under *s*_4_, *s*_3_, *s*_2_, *s*_1_, and *s*_0_ show when segment 4 (head), segment 3, segment 2, segment 1, and segment 0 (tail) gripped the upper side of the step for the first time, respectively. The *red numbers* above the *green lines* correspond to snapshot numbers in Figure 6. The *red lines* are phase differences realized by Kuramoto-PGPE. They remained stationary over time. The *blue lines* are phase differences by SensorCPG-PGPE. Phase relations changed as the robot received different sensor inputs.

The robot using SenseCPG-PGPE achieved longer locomotion distances than those using Kuramoto-PGPE in the experiment of climbing a step because the former switched behaviors between plane locomotion stage and step climbing stage. Figure 15 shows phase differences of oscillators for RTTSs during an episode. The green line under the *h* mark shows when the head segment of a caterpillar controlled by SenseCPG-PGPE first touched the side of the step. The other lines under *s*_4_, *s*_3_, *s*_2_, *s*_1_, and *s*_0_ show when segment 4 (the head), segment 3, segment 2, segment 1, and segment 0 (the tail) gripped the upper side of the step first, respectively. The red lines in Figure 15 denote phase differences realized by Kuramoto-PGPE. The relationship between the phases remained constant for all situations. In locomotion realized by SenseCPG-PGPE, on the other hand, the relationship changed dynamically during climbing and returned to being constant afterward. Because SenseCPG-PGPE was able to switch behaviors for locomotion on a plane and for climbing a step and to separately optimize for each stage, it achieved better performance, whereas Kuramoto-PGPE had to use the same behavior for both stages.

Typically, a CPG-based controller is used to realize periodical behaviors. However, the proposed method achieved reactive behavior, that is, lifting of the head when the head touches the side of a step. Therefore, the method supports the application of CPG-based controllers in broader scenarios, including ones where nonperiodical motion is required.

## Conclusion

In this article, we investigated SenseCPG-PGPE, which is a method for automatically exploring behaviors of a caterpillar-like soft-bodied robot. In this approach, a CPG-based controller consisting of generalized active rotators controls a robot, and the PGPE algorithm optimizes mechanosensory feedback to the CPG-based controller. We showed that this enables the robot to explore various behaviors automatically through body–environment interactions. We also compared several reinforcement learning methods, such as DDPG, TRPO, PPO, and PGPE, and demonstrated that only PGPE, which updates parameters episodically, was able to yield desired behaviors in a caterpillar-like soft-bodied robot. Moreover, the framework automatically designs a feedback controller for a robot with a large number of sensors and actuators. This scalability is important as it becomes possible to implement larger numbers of sensors and actuators in more complex robots. Although SenseCPG-PGPE adopts the CPG-based controller as a key component, it can design a controller that achieves not only steady-state motion, such as straight locomotion, but also nonsteady motion, such as climbing a step, by utilizing mechanosensor information. While we have only applied the proposed method to a caterpillar-like soft-bodied robot, the control target is not limited to such morphology. These methods are also applicable to various soft-bodied robots, such as multilegged soft-bodied robots, because the approach does not make any assumptions about the shape of the robot.

### Future work

There are numerous directions in which to continue developing the proposed framework. First, validation in 3D simulation for generating more diverse and complex behaviors, such as swinging motions, should be conducted. Application to more complex robot shapes, such as quadrupeds and octopi, should also be explored. Testing of the method on a real robot is a crucial next step, although this may require a reduction in learning cost. The embodiment of the nonlinearity of the phase oscillators to the body morphology^28^ may lead to learning cost reduction. Moreover, episode-based reinforcement learning methods other than PGPE should be examined, and conditions required for convergence should be clarified. Furthermore, we believe that the proposed method can contribute to the field of robotics-inspired biology^29^ in two ways. First, we can use our method to investigate the influences of body softness on the behaviors of soft animals. This process will provide a deeper understanding of soft animal behaviors and reveal the affordances and advantages of having a soft body. Second, it may help us to understand the mechanism of multitimescale adaptation of animals, that is, adapting to new situations in various timescales such as adaptation using evolution, learning, and mechanosensory reflexes. We are planning to explore the possibility of introducing a multitimescale-RNN to replace the oscillators, which have a fixed time constant, with more flexible ones to learn multitimescale dynamics, as Yamashita and Tani^30^ demonstrated. By triggering the learning process of RNNs with different time constants, multitimescale adaptation may be achieved. If so, this would contribute to an understanding of the adaptation ability of animals.

## Supplementary Material

Supplemental data

## Supplementary Data

Values of obtained weights: actual values of weights visualized in Discussion section are available at Supplementary Data.
